# The Performance of Artificial Intelligence in Cervical Colposcopy: A Retrospective Data Analysis

**DOI:** 10.1155/2022/4370851

**Published:** 2022-01-05

**Authors:** Yuqian Zhao, Yucong Li, Lu Xing, Haike Lei, Duke Chen, Chao Tang, Xiaosheng Li

**Affiliations:** ^1^Center for Cancer Prevention Research, Sichuan Cancer Hospital and Institute, Sichuan Cancer Center, School of Medicine, University of Electronic Science and Technology of China, Chengdu 610041, China; ^2^Department of Gynecologic Oncology, Chongqing University Cancer Hospital, Chongqing 400030, China; ^3^College of Bioengineering, Chongqing University, Chongqing 400044, China; ^4^Appointment of Follow-up Center, Chongqing University Cancer Hospital, Chongqing 400030, China; ^5^Department of Gynecology, Shenzhen Maternity & Child Healthcare Hospital, Shenzhen 518000, China; ^6^Department of Medical Record Management, Chongqing University Cancer Hospital, Chongqing 400030, China

## Abstract

**Objective:**

We aimed to evaluate the performance of artificial intelligence (AI) system in detecting high-grade precancerous lesions.

**Methods:**

A retrospective and diagnostic study was conducted in Chongqing Cancer Hospital. Anonymized medical records with cytology, HPV testing, colposcopy findings with images, and the histopathological results were selected. The sensitivity, specificity, and areas under the curve (AUC) in detecting CIN2+ and CIN3+ were evaluated for the AI system, the AI-assisted colposcopy, and the human colposcopists, respectively.

**Results:**

Anonymized medical records from 346 women were obtained. The images captured under colposcopy of 194 women were found positive by the AI system; 245 women were found positive either by human colposcopists or the AI system. In detecting CIN2+, the AI-assisted colposcopy significantly increased the sensitivity (96.6% vs. 88.8%, *p*=0.016). The specificity was significantly lower for AI-assisted colposcopy (38.1%), compared with human colposcopists (59.5%, *p* < 0.001) or the AI system (57.6%, *p* < 0.001). The AUCs for the human colposcopists, AI system, and AI-assisted colposcopy were 0.741, 0.765, and 0.674, respectively. In detecting CIN3+, the sensitivities of the AI system and AI-assisted colposcopy were not significantly higher than human colposcopists (97.5% vs. 92.6%, *p*=0.13). The specificity was significantly lower for AI-assisted colposcopy (37.4%) compared with human colposcopists (59.2%, *p* < 0.001) or compared with the AI system (56.6%, *p* < 0.001). The AUCs for the human colposcopists, AI system, and AI-assisted colposcopy were 0.759, 0.674, and 0.771, respectively.

**Conclusions:**

The AI system provided equally matched sensitivity to human colposcopists in detecting CIN2+ and CIN3+. The AI-assisted colposcopy significantly improved the sensitivity in detecting CIN2+.

## 1. Introduction

Cervical cancer is a common malignant tumor among women. According to the estimation of the International Agency for Research on Cancer, there were more than 600,000 new cases worldwide and 340,000 women died from cervical cancer in 2020 [1]. It is well known that persistent infection with high-risk human papillomavirus (HPV) is the cause for cervical cancer and precancerous lesions, and cervical cancer is highly preventable by vaccination of prophylactic HPV vaccine and screening [2].

In recent decades, HPV testing is recommended to be used as a primary screening approach by guidelines. Women with positive screening results of HPV testing and cytology would be referred to colposcopy and biopsy [3]. The pathological diagnosis of the biopsy specimen is the golden standard for the early diagnosis of cervical cancer and precancerous lesions. Hence, the biopsy specimen obtained under colposcopy is essential for the accurate diagnosis. However, the accuracy of colposcopy and biopsy depends on the experience of the colposcopists [4]. The accuracy and reproducibility among different colposcopists and between the colposcopy finding and histopathology confirmed CIN varies greatly [5, 6]. To avoid wasting of health resources caused by overdiagnosis or missing cases, it is imperative to improve the diagnostic accuracy of colposcopy [7].

With the fast development of computing and Internet science, artificial intelligence (AI) has been engaged in the healthcare industry in recent years, especially in the diagnosis of cancers [8–12]. In the field of cervical cancer prevention, efforts have been made in the development of computing scoring systems and artificial intelligence [13–19]. Computing scoring systems involving artificial intelligence were proposed to improve the quality of management of women with abnormal screening results [13, 15]. Computational analysis was involved to improve the accuracy of cytology grading [16–19]. In the year 2020, Xue et al. reported that a colposcopic artificial intelligence auxiliary diagnostic system (CAIADS) for grading colposcopic impressions and guiding biopsies was developed and successfully validated and concluded that CAIADS achieved high sensitivity and comparable specificity to colposcopies interpreted by colposcopists [20]. We are interested in the performance of the AI system that identified the colposcopic images alone or assisted the human colposcopists in detecting high-grade cervical precancerous lesions. In this study, we selected an independent dataset to further evaluate its performance as an independent diagnosis system and as an assisted system.

## 2. Materials and Methods

This was a retrospective, diagnostic study in Chongqing University Cancer Hospital, Chongqing, China. The cytology, HPV testing, colposcopy findings, and histopathological results were collected along with the colposcopy images. The selected records should be cytology abnormal or HPV testing positive, or self-reported symptoms that the gynecologists decided to perform colposcopy examination and biopsy and had colposcopy examination with sequential images for diagnosis and histopathology diagnosis. The images were captured by electronic colposcopy devices (Goldway, China) and were stored in a JPEG format (640 pixels × 480 pixels). The images for each woman included at least five images, which included a preacid image and four postacid images at 60 s, 90 s, 120 s, and 150 s. The personal information of all selected records was fully anonymized. All methods were carried out in accordance with relevant guidelines and regulations. The study was approved by the Research Ethics Committee of Chongqing University Cancer Hospital. The need for informed consent was waived due to the fully anonymized personal information.

### 2.1. Cytology and HPV Testing

The cytology findings of the selected medical records were liquid-based cytology results and were reported according to the 2014 Bethesda nomenclature, including negative for intraepithelial lesion or malignancy (NILM), atypical squamous cells of undetermined significance or worse (ASC-US+), atypical glandular cells (AGC), atypical squamous cells that cannot exclude high-grade squamous intraepithelial lesion (ASC-H), the low-grade squamous intraepithelial lesion (LSIL), the high-grade squamous intraepithelial lesion (HSIL), squamous cell carcinoma (SCC), adenocarcinoma in situ (AIS), and adenocarcinoma (ADC).

HPV testing was performed by the Liferiver genotyping polymerase chain reaction (PCR) test. It detected 13 hrHPV subtypes (HPV 16, 18, 31, 33, 35, 39, 45, 51, 52, 56, 58, 59, and 68). A positive result indicated the detection of any of the high-risk HPV subtype.

### 2.2. Colposcopy and Histopathology

In the colposcopy examination, 5% of acetic acid was applied to the cervix. The colposcopy finding was classified as normal/benign or abnormal (including low-grade, high-grade, and cancer). A punch biopsy was performed if acetowhitening epithelium was observed after the application of the acetic acid. The colposcopy-directed biopsy was performed targeted on each suspected lesion area. If colposcopy impression was normal, HPV testing and/or cytology results, self-reported symptoms, disease history, and benign findings (such as a polyp and condyloma) were taken into consideration for the necessity of performing biopsies or diagnostic excision. Endocervical curettage (ECC) was performed if necessary.

The pathological results of the histological specimens were the golden standard. The final pathological diagnosis for a woman was based on the worst finding from the histopathological slides. All slides were reviewed by pathology experts from the Chongqing University Cancer Hospital.

### 2.3. The AI System

The development and validation of the AI system were reported elsewhere by Xue et al. [11]. The AI system is consisted of a deep learning framework and a risk prediction scoring model. A convolutional neural network (CNN) is trained to crop cervix region from the colposcopy images. The CNN–ResNet-50 [21] is employed as the backbone to identify the cervix bounding box. A fully convolutional network, U-Net5, is adopted in the AI system to perform lesion area segmentation. The cervical images with manual annotation on the lesion areas were used to train and validate the lesion segmentation U-Net. To address the false negative yielded by the deep learning framework, a risk prediction scoring model was designed to optimize the diagnosis by analyzing the cytology or/and HPV testing results. Cases with negative colposcopy but HSIL + cytology and hrHPV 16/18 with LSIL + cytology were suggested to be biopsied. The example pictures of the AI system identify and mark the suggested areas for biopsy and are shown in Supplementary Figure 1.

### 2.4. Statistical Analysis

CIN2+ and CIN3+ were the clinical endpoints for the evaluation, respectively. The finding of the AI system was a dichotomy variable. A positive result of the AI system indicated a low-grade or worse finding under colposcopy. The addition of the AI system to human colposcopists was named “AI-assisted colposcopy.” The AI-assisted colposcopy was a dichotomy variable, and a positive result of AI-assisted colposcopy was defined as either human colposcopists or the AI system finding was positive.

The enrolled medical data were classified by histopathology finding (negative, CIN1, CIN2, and CIN3+), cytology result (NILM, ASC-US, AGC, LSIL, ASC-H, HSIL, and SCC), HPV status (negative, HPV 16/18 positive, or other high-risk subtypes positive), human colposcopists colposcopy finding (normal, LSIL, HSIL, or cancer), the AI system finding, and the AI-assisted colposcopy (negative or positive).

The sensitivity, specificity, positive predictive value (PPV), and negative predictive value (NPV) were evaluated with 95% confidence intervals (CIs) calculated by the Wilson score method. The areas under the curve (AUC) were evaluated. McNemar's test was used to evaluate the differences in sensitivity and specificity between the AI system, AI-assisted colposcopy, and human colposcopists. A *p* value less than 0.05 (two-sided) was considered to be statistically significant. Statistical analyses were conducted with IBM SPSS 21 software (IBM, New York, USA).

## 3. Results

Between January 2019 and October 2019, anonymized medical records of 346 women were obtained. The detailed clinical characteristics of the dataset are given in Table 1. Of 346 women, 214 (61.85%) were cytology abnormal (ASC-US+), 111 (32.08%) were HPV 16/18 positive only, 18 (5.20%) were HPV 16/18 positive and coinfected with other high-risk HPV subtypes, and 99 (28.61%) were other high-risk HPV subtypes positive. Under colposcopy, 183 (52.89%) women were LSIL or worse; the images captured under colposcopy of 194 (56.07%) women were found positive by the AI system; 245 women were found positive either by the human colposcopists or the AI system. In total, 90 women were diagnosed as CIN1, 89 (25.72%, 89/346) were CIN2+, and 81 were CIN3+ (23.41%, 81/346).

The sensitivity, specificity, PPV, NPV, and AUCs of the human colposcopists' findings, AI system, and AI-assisted colposcopy are given in Table 2.

In detecting CIN2+, the sensitivity of the human colposcopists, AI system and AI-assisted colposcopy was 88.8% (95% CI: 80.5%, 93.8%), 95.5% (95% CI: 89.0%, 98.2%), and 96.6% (95% CI: 90.6%, 98.9%), respectively. The sensitivity of the AI system was not significantly higher than human colposcopists (95.5% vs. 88.8%, *p*=0.07). However, the addition of the AI system to the human colposcopists significantly increased the sensitivity (96.6% vs. 88.8%, *p*=0.016). The specificity of the human colposcopists, AI system, and AI-assisted colposcopy was 59.5% (95% CI: 53.4%, 65.4%), 57.6% (95% CI: 51.5%, 63.5%), and 38.1% (95% CI: 32.4%, 44.2%), respectively. The specificity was significantly lower for the AI-assisted colposcopy, compared with human colposcopists (38.1% vs. 59.5%, *p* < 0.001) or compared with the AI system (38.1% vs. 57.6%, *p* < 0.001). For PPVs and NPVs, no significant statistical difference was detected between human colposcopists and the AI system (PPV: 43.2% vs. 43.8%, *p*=0.90; NPV: 93.9% vs. 97.4%, *p*=0.13) or between human colposcopists and AI-assisted colposcopy (PPV: 43.2% vs. 38.1%, *p*=0.09; NPV: 93.9% vs. 97.0%, *p*=0.25). The AUCs for the human colposcopists, AI system, and AI-assisted colposcopy were 0.741 (95% CI: 0.686, 0.797), 0.765 (95% CI: 0.715, 0.816), and 0.674 (95% CI: 0.616, 0.731), respectively.

In detecting CIN3+, the sensitivity of the human colposcopists, AI system, and AI-assisted colposcopy was 92.6% (95% CI: 84.8%, 96.6%), 97.5% (95% CI: 91.4%, 99.3%), and 97.5% (95% CI: 91.4%, 99.3%). The sensitivity of the AI system and AI-assisted colposcopy was not significantly higher than human colposcopists (97.5% vs. 92.6%, *p*=0.13). The specificity of the human colposcopists, AI system, and AI-assisted colposcopy was 59.2% (95% CI: 53.2%, 65.0%), 56.6% (95% CI: 50.6%, 62.4%), and 37.4% (95% CI: 31.8%, 43.3%), respectively. The specificity was significantly lower for AI-assisted colposcopy, compared with human colposcopists (37.4% vs. 59.2%, *p* < 0.001) or compared with the AI system (37.4% vs. 56.6%, *p* < 0.001). For PPVs and NPVs, no significant statistical difference was detected between human colposcopists and the AI system (PPV: 41.0% vs. 40.7%, *p*=0.96; NPV: 96.3% vs. 98.7%, *p*=0.18) or between human colposcopists and the AI-assisted colposcopy (PPV: 41.0% vs. 32.2%, *p*=0.06; NPV: 96.3% vs. 98.0%, *p*=0.43). The AUCs for the human colposcopists, AI system, and AI-assisted colposcopy were 0.759 (95% CI: 0.706, 0.812), 0.674 (95% CI: 0.616, 0.733), and 0.771 (95% CI: 0.721, 0.820), respectively.

## 4. Discussion

In the presented study, we further validated the accuracy of the colposcopic deep learning auxiliary diagnosis system developed by Xue et al. The results showed that the AI system alone was accurate as of the human colposcopists in detecting high-grade precancerous lesions of the cervix with comparable sensitivity and specificity. The addition of the AI system to the human colposcopists could improve the sensitivity of detecting histopathological confirmed CIN2+, although with a lower specificity.

Colposcopy is a real-time visualization and assessment instrument of the cervix for the detection of CINs and invasive cancer. The accuracy of colposcopy and colposcopy-guided biopsy in detecting high-grade CIN and cervical cancer has been a concern for decades. It has been well documented that colposcopic assessment and biopsy were less reproducible and could miss a substantial proportion of prevalent high-grade CIN, and the false negative rate ranges from 13% to 69% [22–25]. To minimize the potential harm caused by the colposcopy and biopsy, it was suggested that the colposcopy should be performed by a well-trained, knowledgeable provider to reduce inaccurate diagnosis and resultant inappropriate management [26]. However, in real-world clinical practice, the countries and areas that suffered from the heavy disease burden of cervical cancer were usually at a shortage of experienced colposcopists. To improve the sensitivity of colposcopy-guided biopsy, some suggested taking a multibiopsy and random biopsy from the normal appearing quadrants [27–29]. However, a widely adopted biopsy guideline is absent hitherto.

As computer science and technology are developing rapidly, the advantages of AI are at recognizing complex patterns in images and transforming the image interpretation from a qualitative and subjective task to one that is quantifiable and effortlessly reproducible [30]. The problem that being short of well-trained personnel seemed to be possible to be solved within a shorter time interval. The application of artificial intelligence for medical services has become promising for cancer and precancerous lesions screening [30]. To meet the need of improving the quality of colposcopy and biopsy, especially in low and middle-income countries, Xue et al. developed and validated a colposcopic deep learning auxiliary diagnosis system. In the previous results reported by Xue et al., the AI system achieved a high agreement (82.2%) for grading colposcopic impressions with the pathological gold standard (kappa 0.750). However, the observation agreement between the AI system grading and histopathological findings was 66.9% for HSIL. Since the task for the colposcopy examination is to decide whether to take a biopsy or not and to locate the suspicious lesions for detecting underlying cervical precancerous lesions for subsequent treatment, the AI system-graded HSIL finding seemed not to be a practical threshold for biopsy. In their validation set, if the biopsy threshold is set at low-grade or worse colposcopy findings, the sensitivity for the analysis of images by the AI method was 87.3% (95% CI: 85.5%, 88.9%) and the specificity was 48.9% (95% CI: 46.8%, 50.9%). In our data, the sensitivity for detecting CIN2+ by the AI system was 95.5% (95% CI: 89.0%, 98.2%) and the specificity was 57.6% (95% CI: 51.5%, 63.5%), respectively. The sensitivity for detecting CIN3+ by the AI system was numerically higher as 97.5% (95% CI: 91.4%, 99.3%). Xue et al. did not report the accuracy of adding CAIADS to the human colposcopists, instead of presenting the diagnostic performance of CAIADS and colposcopists separately, because the main task for the previous study was to construct an accurate AI method. However, for clinical implementation, it may not be possible to make the decision of biopsy based on the AI system alone, although it showed comparable sensitivity and specificity to the human colposcopists. Our data implied that the scenario of combining the AI system and the human colposcopists were practical, since in a population with a high risk of cervical cancer and precancerous lesions identified by HPV testing and/or cytology, a relatively higher sensitivity with a loss of specificity may be tolerable in clinical practice.

Our study further compared the performance of the AI system with human colposcopists and evaluated the AI-assisted colposcopy in a practical clinical condition. The results suggested in resource-limited areas that lack well-trained, knowledgeable colposcopy providers but bear the heavy disease burden of cervical cancer; the AI system may be useful for assisting the biopsy procedure and for training young colposcopists. The limitation of this study was the single-center and retrospective design. The disagreement between the AI system and human colposcopists could not be addressed by the present study if extra biopsy was suggested by the AI system. A prospective study is necessary to further validate the predictive performance of the AI system and the AI-assisted colposcopy.

In conclusion, our study indicates that the analysis on the images of the AI system provided equally matched sensitivity to the human colposcopists in detecting CIN2+ and CIN3+. The AI-assisted colposcopy significantly improved the sensitivity in detecting CIN2+.

## Figures and Tables

**Table 1:** Clinical results of the enrolled medical records.

	Total (*N*)	Histopathology diagnosis									
		Normal, *n* (%)		CIN1, *n* (%)		CIN2, *n* (%)		CIN3, *n* (%)		Cancer, *n* (%)	
Cytology											
NILM	132	89	67.4	38	28.8	1	0.8	1	0.8	3	2.3
ASC-US	98	61	62.2	29	29.6	2	2.0	2	2.0	4	4.1
AGC	6	2	33.3	1	16.7	0	0.0	0	0.0	3	50.0
LSIL	32	10	31.3	16	50.0	2	6.3	2	6.3	2	6.3
ASC-H	9	3	33.3	5	55.6	1	11.1	0	0.0	0	0.0
HSIL	17	2	11.8	1	5.9	2	11.8	4	23.5	8	47.1
SCC	52	0	0.0	0	0.0	0	0.0	1	1.9	51	98.1
HPV test											
Negative	113	72	63.7	33	29.2	3	2.7	1	0.9	4	3.5
HPV 16/18 positive	129	43	33.3	23	17.8	4	3.1	5	3.9	54	41.9
Other high-risk subtypes positive	99	48	48.5	33	33.3	1	1.0	4	4.0	13	13.1
Colposcopy findings by the human colposcopists											
Normal	163	107	65.6	46	28.2	4	2.5	2	1.2	4	2.5
LSIL	89	51	57.3	29	32.6	2	2.2	3	3.4	4	4.5
HSIL	34	8	23.5	14	41.2	1	2.9	2	5.9	9	26.5
Cancer	60	1	1.7	1	1.7	1	1.7	3	5.0	54	90.0
AI system findings^*∗*^											
Negative	152	121	79.6	27	17.8	2	1.3	0	0.0	2	1.3
Positive	194	46	23.7	63	32.5	6	3.1	10	5.2	69	35.6
AI-assisted finding^†^											
Negative	101	85	84.2	13	12.9	1	1.0	0	0.0	2	2.0
Positive	245	82	33.5	77	31.4	7	2.9	10	4.1	69	28.2
Total	346	167	48.3	90	26.0	8	2.3	10	2.9	71	20.5

^*∗*^A positive finding by the AI system indicated the finding of the images was classified as positive by the AI system alone. ^†^A positive finding by the AI-assisted finding indicated the finding of the images was classified as positive either of the AI system or human colposcopists or both. AI, artificial intelligence; ASC-US, atypical squamous cells of undetermined significance; ASC-H, atypical squamous cells that cannot exclude high-grade squamous intraepithelial lesion; AGC, atypical glandular cells; CIN, cervical intraepithelial neoplasia; HPV, human papillomavirus; HSIL, high-grade squamous intraepithelial lesion; LSIL, low-grade squamous intraepithelial lesion; NILM, negative for intraepithelial lesion or malignancy; SCC, squamous cell carcinoma.

**Table 2:** The sensitivity, specificity, positive predictive value, negative predictive value, and AUCs of the human colposcopists, AI system, and AI-assisted colposcopy.

	Sensitivity % (95% CI)	Specificity % (95% CI)	Positive predictive value % (95% CI)	Negative predictive value % (95% CI)	AUC (95% CI)
CIN2+					
Human colposcopists	88.8 (80.5, 93.8)	59.5 (53.4, 65.4)	43.2 (36.2, 50.4)	93.9 (89.1, 96.6)	0.741 (0.686, 0.797)
AI system	95.5 (89.0, 98.2)	57.6 (51.5, 63.5)	43.8 (37.0, 50.9)	97.4 (93.4, 99.0)	0.765 (0.715, 0.816)
AI-assisted colposcopy	96.6 (90.6, 98.9)	38.1 (32.4, 44.2)	35.1 (29.4, 41.3)	97.0 (91.6, 99.0)	0.674 (0.616, 0.731)

CIN3+					
Human colposcopists	92.6 (84.8, 96.6)	59.2 (53.2, 65.0)	41.0 (34.1, 48.2)	96.3 (92.2, 98.3)	0.759 (0.706, 0.812)
AI system	97.5 (91.4, 99.3)	56.6 (50.6, 62.4)	40.7 (34.1, 47.8)	98.7 (95.3, 99.6)	0.674 (0.616, 0.733)
AI-assisted colposcopy	97.5 (91.4, 99.3)	37.4 (31.8, 43.3)	32.2 (26.7, 38.3)	98.0 (93.1, 99.5)	0.771 (0.721, 0.820)

AI, artificial intelligence; AUC, areas under the curve; CIN, cervical intraepithelial neoplasia; 95% CI, 95% confidence intervals.

## Data Availability

The datasets used and/or analyzed during the present study are available from the corresponding author upon request.

## Supplementary Materials

Supplementary Materials The performance of artificial intelligence in cervical colposcopy: a retrospective data analysis.
