# Supplementary material for: The Performance of Artificial Intelligence in Cervical Colposcopy: A Retrospective Data Analysis
Source: J Oncol. 2022 Jan 5;2022:4370851. doi: 10.1155/2022/4370851 (PMC8754610; doi:10.1155/2022/4370851)
Supplement: Supplementary Materials — The performance of artificial intelligence in cervical colposcopy: a retrospective data analysis. [file 4370851.f1.docx]

**Supplementary appendix**

Supplement to: The performance of artificial intelligence in cervical colposcopy: a retrospective data analysis.

**Supplementary** **Figure 1.** Example of the AI-system identify and mark the suggested areas for biopsy**.**


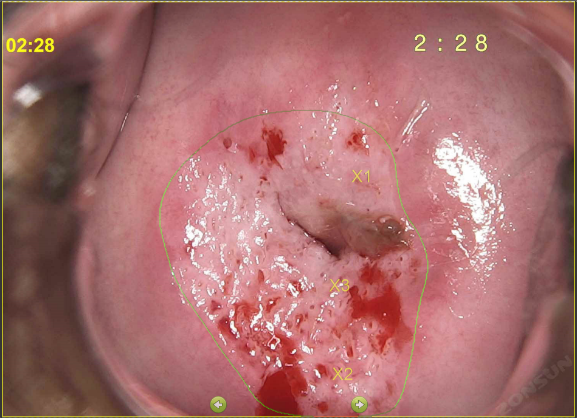


B


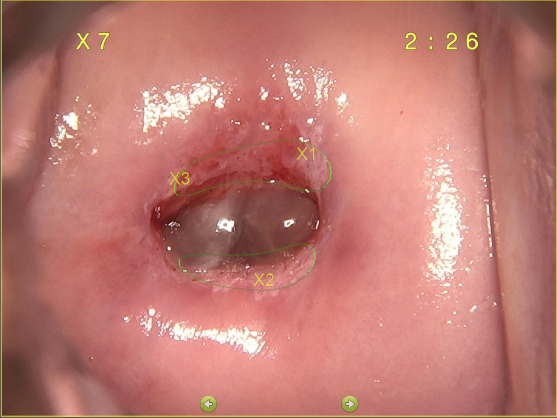


A

**Cervix image annotation examples.** The yellow contour annotates the images boundaries of distinct acetowhitening lesions. The tag X mark the suggested areas for biopsy. The colposcopy image A was identified as low-grade lesion, image B was high-grade lesion by the AI-system respectively. The final pathology diagnosis were in accordance with the colposcopic findings by the AI-system.
